# Association of cardiometabolic index with all-cause and cause-specific mortality among U.S. adult population: A longitudinal cohort study

**DOI:** 10.1097/MD.0000000000043532

**Published:** 2025-07-25

**Authors:** Caijuan Huang, Lele Chen

**Affiliations:** a Department of Hematology, The First Affiliated Hospital, and College of Clinical Medicine of Henan University of Science and Technology, Luoyang, China; b Department of Vascular Surgery of Xincai County People's Hospital, Zhumadian, China.

**Keywords:** all-cause mortality, cardiometabolic index, cox proportional hazards model, National Health and Nutrition Examination Survey, specific-cause mortality

## Abstract

Currently, although some studies have explored the association between the cardiometabolic index (CMI) and mortality, for the adult population in the United States, detailed and systematic research on the link between CMI and both all-cause and cause-specific mortality remains insufficient. This study included a general population of 12,845 individuals with complete data from National Health and Nutrition Examination Survey 2005 to 2018. Mortality data were extracted from the National Death Index up to December 31, 2019. Restricted cubic spline analysis was used to explore the nonlinear association between CMI and mortality. Additionally, stratified analyses, interaction tests, and several sensitivity analyses were conducted to assess the robustness of the results. Over an average follow-up period of 7.7 years, there were 1388 deaths from all causes, including 322 cancer deaths and 413 cardiovascular disease (CVD) deaths. Weighted Kaplan–Meier survival analysis showed an increasing incidence of all-cause mortality, cancer mortality, and CVD mortality from the lowest to the highest quartiles of CMI, with Log-rank *P* < .001, Log-rank *P* = .014, and Log-rank *P* < .001, respectively. After full adjustment, weighted Cox regression demonstrated a nearly linear increase in CVD mortality with increasing CMI; compared to the referent quartile, the hazard ratios for Quartile 2 were 1.69 (95% confidence interval [CI]: 1.16–2.46; *P* = .01), Quartile 3 were 1.69 (95% CI: 1.12–2.55; *P* = .01), and Quartile 4 were 1.77 (95% CI: 1.16–2.70; *P* = .01) (*P* for trend = .04). However, the association between CMI and all-cause mortality as well as cancer mortality was not significant based on COX regression (all *P* > .05). Restricted cubic spline analysis revealed a significant linear relationship between CMI and CVD mortality (nonlinear *P* = .051). Interaction analysis confirmed consistent associations between CMI and CVD mortality across all subgroups (all *P* interaction > .05). Additionally, sensitivity analyses confirmed the robustness of the results mentioned above. CMI demonstrates an almost linear increase in CVD mortality among the general adult population in the United States, while showing no association with all-cause and cancer mortality. This indicates a more direct impact of CMI on heart health and the development of CVD.

## 1. Introduction

Obesity is a major global issue that has attracted significant attention worldwide. With changes in lifestyle, dietary patterns, and lack of exercise, obesity is becoming increasingly serious on a global scale.^[1,2]^ According to the 2023 edition of the “World Obesity Map,” it is predicted that by 2035, there will be over 4 billion people globally classified as obese or overweight, accounting for 51% of the global population.^[3]^ Obesity not only increases the risk of developing chronic diseases such as cardiovascular disease (CVD), diabetes, and certain types of cancer, but also has a negative impact on individuals’ quality of life and life expectancy.^[4,5]^

Numerous indicators reflect obesity. For a long time, body mass index (BMI) has been used to assess obesity and has some predictive value for various chronic diseases and mortality. However, recent epidemiological evidence has revealed drawbacks of BMI in reflecting mortality outcomes, leading to the emergence of the “obesity paradox” concept.^[6]^ In several studies, being overweight has been associated with an increased risk of mortality,^[7]^ while some researchers have found that overweight individuals have the lowest mortality rates, with mortality decreasing as BMI increases.^[8,9]^ Fortunately, in recent years, some promising obesity indicators have been developed. The visceral adiposity index (VAI), as a useful indicator of fat distribution and function, combines anthropometric data and lipid profiles, making it a reliable predictor of visceral dysfunction.^[10,11]^ Since its introduction, VAI has been found to exhibit a J-shaped relationship with all-cause mortality among elderly Americans and patients with chronic kidney disease^[12,13]^; it also shows a reverse “U” relationship with coronary heart disease risk and an “L”-shaped association with coronary heart disease mortality in the adult American population.^[14]^

In 2015, Wakabayashi et al^[15]^ first proposed the cardiometabolic index (CMI), which combines waist-to-height ratio (WHtR) and the ratio of triglycerides to high-density lipoprotein cholesterol (TG/HDL) to comprehensively reflect abdominal obesity and lipid abnormalities. Compared to traditional anthropometric measures, CMI more accurately predicts metabolic abnormalities in the body.^[16,17]^ To date, CMI has been shown to be closely associated with CVD, liver and kidney diseases, psychiatric disorders, and renal cancer.^[15,18–27]^ However, despite the confirmation of the association of CMI with cardiovascular health, cancer, and metabolic diseases, there is still a lack of relatively reliable epidemiological evidence regarding the related risks of mortality and the severity of diseases.

To address this knowledge gap, we conducted this longitudinal cohort study. Survival status among different quartiles of CMI was estimated through Kaplan–Meier survival analysis. A weighted multivariable Cox proportional hazards regression model was constructed to determine the relationship between CMI and the risk of all-cause mortality, cancer-specific mortality, and CVD-specific mortality. A restricted cubic spline regression model was used to explore the dose–response relationship of this association. Additionally, subgroup and interaction analyses were employed to identify potentially vulnerable populations. Finally, several sensitivity analyses were performed to validate the robustness of the conclusions.

## 2. Methods

### 2.1. Data source

**Figure 1.**
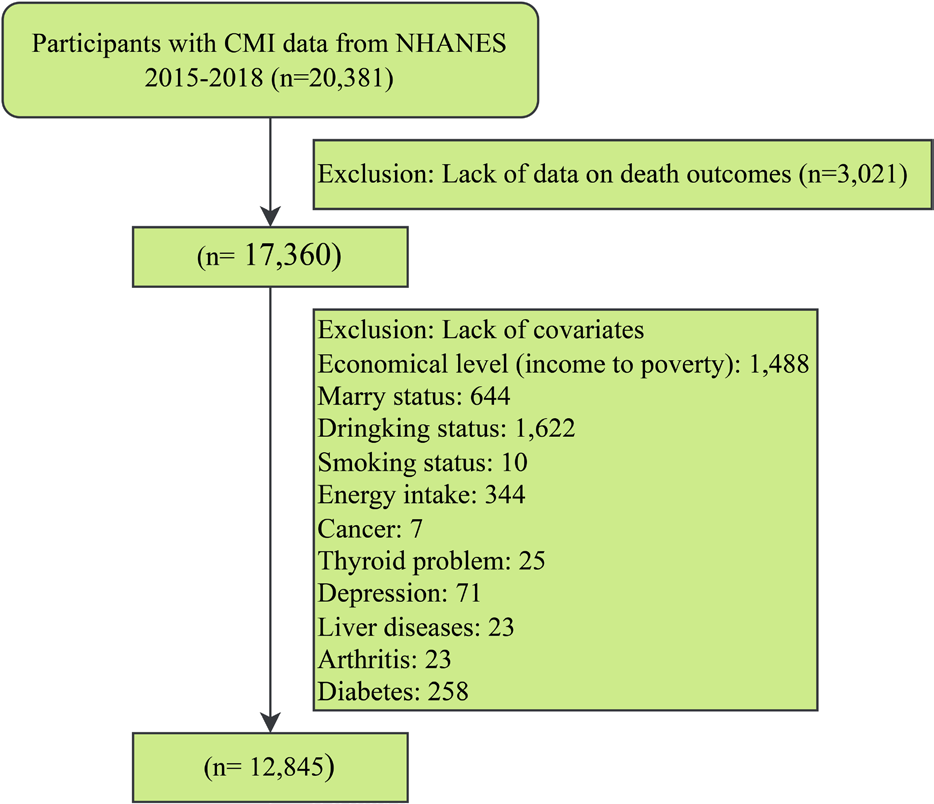
Flow chart for inclusion of participants. CMI = cardiometabolic index; NHANES = National Health and Nutrition Examination Survey.

The National Health and Nutrition Examination Survey (NHANES) is a series of complex, stratified, multistage, continuous, nationally representative surveys designed to investigate the health and nutritional status of the non-institutionalized civilian population in the United States. This cross-sectional study involved participants from 7 cycles of NHANES spanning from 2005 to 2018. Across the 7 cycles, a total of 70,190 participants were initially involved, with subsequent exclusions made for individuals who: (1) were missing any of the 4 metrics required for calculating CMI (height, waist circumference [WC], HDL, TG) (N = 49,809); (2) lacked recorded vital status data (N = 3021); and (3) lacked covariate data (N = 4515). Ultimately, 12,845 eligible participants were included (Fig. 1). The survey obtained ethical approval from the Institutional Review Board of the National Center for Health Statistics, and written informed consent was provided by all participants (https://wwwn.cdc.gov/nchs/nhanes/Default.aspx).

### 2.2. CMI

According to Wakabayashi et al,^[15]^ the calculation formula for CMI is as follows: CMI = (TG × WHtR)/(HDL-C); where WHtR = WC/height. Specifically, TG and HDL-C were obtained from laboratory measurements (MEC); height (in cm), and WC were recorded during participant interviews.

### 2.3. Confirming the death status

We obtained mortality certificate data from the National Death Index (NDI). Using a series of identification numbers, NHANES survey data was linked to NDI data through probabilistic matching to determine participants’ survival status. Participants for whom NDI matching failed were assumed to be alive.^[28,29]^ The follow-up endpoint time was determined by the date of death or December 31, 2019 (whichever came first), and follow-up duration was calculated in years. Disease-specific mortality was determined based on the guidelines of the Tenth Revision of the International Classification of Diseases. Cancer mortality: Deaths caused by Malignant neoplasms (C00-C97).^[30]^ CVD mortality: deaths due to diseases of the heart (I00–I09, I11, I13, I20–I51) and cerebrovascular diseases (I60–I69).

### 2.4. Covariates

During the survey, participants self-reported their age (continuous), gender (male/female), race/ethnicity (Mexican American, African American, Non-Hispanic White, Other Hispanic, Other including Multiracial), education level (below college, college and above), and marital status (divorced/separated/widowed, married/cohabiting, single). Factors such as family income-to-poverty ratio (<1.3, 1.3–3.5, >3.5), lifestyle habits including alcohol consumption (never, former, current), smoking (never, former, current), and total energy intake (24-hour dietary recall) were also considered. Additionally, comorbidities were included in the model, such as cancer, liver disease, CVDs (such as coronary artery disease, congestive heart failure, myocardial infarction, stroke, and angina), diabetes, arthritis, thyroid disease, and depression. Using antidiabetic medication or being informed by a doctor of having diabetes was defined as having diabetes. Depression was determined based on the Patient Health Questionnaire-9, with a total score ≥ 10 defined as depression based on previous studies.^[31–33]^

### 2.5. Statistical analysis

NHANES adopts a complex sampling design, thus this study employs Mobile Examination Center (MEC) weights (1/7 × WTMEC2YR) for subsequent analysis. Baseline characteristics between groups were compared based on CMI quartiles, with continuous variables analyzed using one-way ANOVA or Kruskal–Wallis test and results presented as mean or median (IQR), respectively. Categorical variables were assessed using chi-square (*χ*^2^) tests and expressed as case numbers (n) and percentages (%). Kaplan–Meier survival curves and log-rank tests were utilized to determine mortality risk across CMI quartile groups. Multivariable Cox regression models were constructed to estimate hazard ratios (HRs) and 95% confidence intervals (CIs) for all-cause mortality and cause-specific mortality associated with CMI. Model 0 did not consider confounding factors; Model 1 adjusted for gender, age, race, and education; Model 2 further adjusted for socioeconomic status, marital status, smoking, alcohol consumption, and energy intake; Model 3 additionally adjusted for CVD, cancer, liver disease, diabetes, arthritis, thyroid disease, and depression. The “car” package in R was used to diagnose multicollinearity among model variables, and all variance inflation factors in this study were <2. Schoenfeld residuals were used to test the proportional hazards assumption. Median values of each quartile group replaced the original variables in the Cox regression model to calculate trend *P*-values. Restricted cubic splines at 3 nodes (percentile 10, percentile 50, and percentile 90) were employed to assess the dose–response relationship between continuous CMI and mortality risk. Subgroup analyses were conducted, and effect modification by potential covariates was explored using likelihood ratio tests.

Several sensitivity analyses were performed: (1) excluding participants who died within the 2-year follow-up period; (2) not considering weights; (3) utilizing the “mice” package in R to conduct multiple imputation for missing covariates. This study was conducted using R version 4.3.1 (http://www.r-project.org), and a *P*-value < .05 was considered statistically significant.

### 2.6. Ethical approval

The survey received ethical approval from the Institutional Review Board of the National Center for Health Statistics, and all participants provided written informed consent (Ethics Review Board Approval | National Health and Nutrition Examination Survey | CDC).

## 3. Results

### 3.1. Baseline characteristics

After stringent screening, a total of 12,845 participants were included in the study (Fig. 1). Weighted, this represents approximately 80 million U.S. adults. Over a mean follow-up period of 7.7 years, there were 1388 deaths from all causes, with a weighted mortality rate of 7.41%. Among these, there were 322 deaths attributed to cancer and 413 deaths attributed to CVD. Baseline characteristics of participants stratified by CMI quartiles are presented in Table 1. Compared to the lowest CMI quartile group, the highest CMI quartile group had a younger age, higher mortality rate, more males, and significantly different racial composition. Additionally, they had lower educational levels, more were in a partnered or married status, had poorer economic status, and a higher prevalence of past alcohol consumption and smoking. Furthermore, in terms of comorbidities, participants in the highest CMI quartile group had significantly higher rates of chronic diseases, depression, arthritis, and thyroid disease compared to those in the lowest quartile group.

**Table 1:** Weighted baseline characteristics of the eligible participants.

Characteristics	Total (0.016–2.22) (N = 12,845)	Quartile 1 (0.016–0.094) (N = 3184)	Quartile 2 (0.094–0.14) (N = 3232)	Quartile 3 (0.14–0.215) (N = 3224)	Quartile 4 (0.215–2.22) (N = 3205)	*P* value
Follow-up time (years), mean (SE)	7.77 (0.10)	7.39 (0.14)	7.81 (0.13)	7.98 (0.13)	7.94 (0.12)	<.001
Age (year), mean (SE)	47.49 (0.28)	43.69 (0.46)	47.02 (0.41)	48.91 (0.37)	50.68 (0.41)	<.0001
State of survival, n (%)						<.0001
Survival	11,507 (92.59)	2996 (96.02)	2912 (93.25)	2837 (90.99)	2762 (89.78)	
Death	1338 (7.41)	188 (3.98)	320 (6.75)	387 (9.01)	443 (10.22)	
Sex, n (%)						<.0001
Female	6354 (49.82)	1817 (57.86)	1611 (50.76)	1565 (48.59)	1361 (41.45)	
Male	6491 (50.18)	1367 (42.14)	1621 (49.24)	1659 (51.41)	1844 (58.55)	
Race, n (%)						<.0001
Mexican American	1965 (7.96)	292 (5.18)	457 (7.63)	587 (9.58)	629 (9.73)	
Non-Hispanic Black	2557 (9.87)	911 (14.23)	738 (10.81)	558 (8.69)	350 (5.38)	
Non-Hispanic White	5893 (70.56)	1351 (68.32)	1441 (71.03)	1450 (68.98)	1651 (74.02)	
Other Hispanic	1183 (5.00)	232 (4.52)	292 (4.81)	338 (5.73)	321 (5.00)	
Other race – including multi-racial	1247 (6.60)	398 (7.75)	304 (5.72)	291 (7.02)	254 (5.88)	
Education attainment, n (%)						<.0001
Less than college	5918 (38.10)	1160 (30.18)	1444 (37.20)	1603 (42.15)	1711 (43.59)	
College or higher	6927 (61.90)	2024 (69.82)	1788 (62.80)	1621 (57.85)	1494 (56.41)	
Marital status, n (%)						<.0001
Never married	2267 (17.29)	787 (21.59)	602 (17.85)	485 (16.44)	393 (12.91)	
Divorced/separated/widowed	2789 (18.04)	581 (14.85)	710 (18.81)	752 (20.03)	746 (18.71)	
Married/living with a partner	7789 (64.67)	1816 (63.56)	1920 (63.33)	1987 (63.53)	2066 (68.38)	
Economical level (poverty), n (%)						<.0001
<1.3	3847 (19.85)	826 (17.24)	909 (18.67)	1008 (21.93)	1104 (21.87)	
1.3–3.5	4931 (36.19)	1200 (33.75)	1236 (35.39)	1278 (38.86)	1217 (37.04)	
>3.5	4067 (43.96)	1158 (49.02)	1087 (45.94)	938 (39.22)	884 (41.09)	
Drinking status, n (%)						<.0001
Never	1651 (10.05)	407 (9.84)	410 (10.31)	415 (10.08)	419 (9.96)	
Former	2091 (13.22)	364 (8.94)	459 (11.53)	596 (15.58)	672 (17.28)	
Now	9103 (76.73)	2413 (81.22)	2363 (78.16)	2213 (74.34)	2114 (72.75)	
Smoking status, n (%)						<.0001
Never	6953 (54.17)	1975 (61.48)	1803 (55.74)	1679 (51.28)	1496 (47.50)	
Former	3226 (25.80)	643 (21.61)	758 (24.31)	839 (26.29)	986 (31.37)	
Now	2666 (20.03)	566 (16.92)	671 (19.95)	706 (22.43)	723 (21.14)	
Total energy intake, mean (SE)	2195.60 (11.49)	2176.29 (19.18)	2196.87 (24.97)	2175.55 (22.59)	2234.39 (23.92)	.19
Cancer, n (%)						<.001
No	11,663 (90.50)	2947 (91.66)	2957 (91.22)	2935 (91.17)	2824 (87.84)	
Yes	1182 (9.50)	237 (8.34)	275 (8.78)	289 (8.83)	381 (12.16)	
Thyroid problem, n (%)						.02
No	11,522 (89.27)	2904 (90.38)	2915 (89.83)	2872 (89.30)	2831 (87.45)	
Yes	1323 (10.73)	280 (9.62)	317 (10.17)	352 (10.70)	374 (12.55)	
Liver diseases, n (%)						<.0001
No	12,334 (96.28)	3104 (97.85)	3130 (96.87)	3075 (95.70)	3025 (94.53)	
Yes	511 (3.72)	80 (2.15)	102 (3.13)	149 (4.30)	180 (5.47)	
Arthritis, n (%)						<.0001
No	9321 (73.81)	2592 (81.91)	2419 (76.54)	2248 (71.14)	2062 (64.88)	
Yes	3524 (26.19)	592 (18.09)	813 (23.46)	976 (28.86)	1143 (35.12)	
CVD, n (%)						<.0001
No	11,473 (91.33)	3040 (95.99)	2931 (93.02)	2857 (90.52)	2645 (85.38)	
Yes	1372 (8.67)	144 (4.01)	301 (6.98)	367 (9.48)	560 (14.62)	
Diabetes, n (%)						<.0001
No	11,106 (90.00)	3056 (97.17)	2952 (94.78)	2710 (88.60)	2388 (78.72)	
Yes	1739 (10.00)	128 (2.83)	280 (5.22)	514 (11.40)	817 (21.28)	
Depression, n (%)						<.0001
No	11,800 (92.98)	2982 (94.65)	3029 (94.51)	2954 (92.03)	2835 (90.52)	
Yes	1045 (7.02)	202 (5.35)	203 (5.49)	270 (7.97)	370 (9.48)	

CVD = cardiovascular disease, poverty = ratio of household income-to-poverty level, SD = standard deviation.

### 3.2. The relationship between CMI and survival status

**Figure 2.**
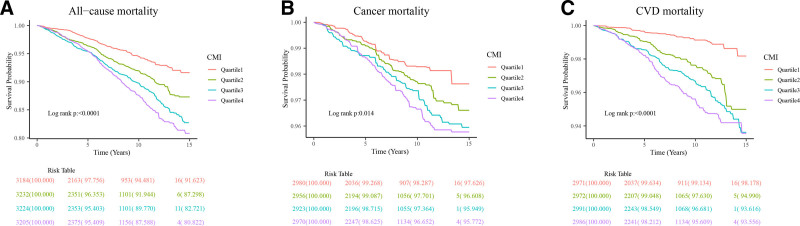
Kaplan–Meier survival analysis for all-cause mortality and cause-specific mortality among the 4 quartile groups. (A) All-cause mortality; (B) Cancer mortality; (C) CVD mortality. CMI = cardiometabolic index, CVD = cardiovascular disease.

The results of the Kaplan–Meier survival analysis are shown in Figure 2. Quartile classification of CMI was associated with lower all-cause and cause-specific survival rates in individuals with high levels of CMI (Log-rank *P* < .001, Log-rank *P* = .014, Log-rank *P* < .001, respectively) (Fig. 2A–C).

Table 2 describes the association of continuous or categorized CMI with the risk of mortality. In this study, Schoenfeld residuals tests suggested that all models passed the proportional hazards assumption. When no confounding factors were adjusted (Model 0), Cox regression estimated that continuous CMI significantly increased the risk of all-cause mortality, cancer mortality, and CVD mortality (all *P* < .01). As adjustments were gradually made, these associations significantly weakened. After full adjustment (Model 3), continuous CMI was only significantly associated with the risk of CVD mortality (HR: 2.05, 95% CI: 1.01–4.14, *P* = .03). When quartile classification of CMI was included in the model, without adjustments (Model 0), compared to Quartile 1, participants in Quartiles 3 to 4 had a significantly increased risk of all-cause mortality, cancer mortality, and CVD mortality (all *P* < .05); after adjusting for demographic factors, lifestyle, chronic diseases, depression, and others (Model 3), CMI was linearly associated only with CVD mortality. Specifically, compared to Quartile 1, the risk of CVD mortality was significantly increased in Quartile 2 (HR: 1.75, 95% CI: 1.19–2.58, *P* = .005), Quartile 3 (HR: 1.89, 95% CI: 1.27–2.83, *P* = .002), and Quartile 4 (HR: 2.09, 95% CI: 1.36–3.20, *P* < .001) (P trend = 0.003).

**Table 2:** Estimated HRs of continuous or categorical CMI with all-cause mortality and cause-specific mortality.

All-Cause mortality								
	Model 0		Model 1		Model 2		Model 3	
	HR (95% CI)	*P*	HR (95% CI)	*P*	HR (95% CI)	*P*	HR (95% CI)	*P*
Quartile 1	Ref		Ref		Ref		Ref	
Quartile 2	1.59 (1.27, 2.00)	<.0001	1.13 (0.90, 1.43)	.29	1.11 (0.88, 1.39)	.38	1.06 (0.85, 1.32)	.62
Quartile 3	2.07 (1.66, 2.57)	<.0001	1.29 (1.01, 1.65)	.04	1.18 (0.93, 1.49)	.17	1.08 (0.85, 1.37)	.54
Quartile 4	2.36 (1.87, 2.98)	<.0001	1.33 (1.05, 1.69)	.02	1.20 (0.95, 1.51)	.12	1.01 (0.79, 1.27)	.96
*P* for trend		<.0001		.01		.1		.91
Continuous	3.39 (2.38, 4.84)	<.0001	2.10 (1.40, 3.15)	<.001	1.69 (1.12, 2.54)	.01	1.12 (0.70, 1.80)	.63
Cancer mortality								
Quartile 1	Ref		Ref		Ref		Ref	
Quartile 2	1.40 (0.90, 2.19)	.13	0.96 (0.62, 1.50)	.86	0.95 (0.62, 1.46)	.80	0.98 (0.63, 1.51)	.91
Quartile 3	1.77 (1.14, 2.74)	.01	1.06 (0.66, 1.72)	.80	0.94 (0.59, 1.50)	.80	0.95 (0.59, 1.53)	.84
Quartile 4	1.95 (1.20, 3.17)	.01	1.09 (0.67, 1.77)	.73	0.99 (0.62, 1.58)	.96	0.98 (0.59, 1.64)	.94
*P* for trend		.003		.59		.99		.94
Continuous	2.26 (1.29, 3.94)	.004	1.22 (0.54, 2.76)	.63	0.94 (0.41, 2.16)	.88	0.91 (0.38, 2.19)	.84
CVD mortality								
Quartile 1	Ref		Ref		Ref		Ref	
Quartile 2	3.01 (2.05, 4.42)	<.0001	1.82 (1.22, 2.70)	.003	1.81 (1.22, 2.69)	.003	1.75 (1.19, 2.58)	.005
Quartile 3	4.10 (2.80, 6.00)	<.0001	2.24 (1.49, 3.35)	<.0001	2.04 (1.36, 3.06)	<.001	1.89 (1.27, 2.83)	.002
Quartile 4	4.99 (3.32, 7.49)	<.0001	2.58 (1.69, 3.95)	<.0001	2.35 (1.53, 3.61)	<.0001	2.09 (1.36, 3.20)	<.001
*P* for trend		<.0001		<.0001		<.001		.003
Continuous	4.40 (2.60, 7.43)	<.0001	3.66 (2.04, 6.54)	<.0001	3.35 (1.84, 6.09)	<.0001	2.05 (1.01, 4.14)	.03

*Notes*: Model 0, no confounder were adjusted; Model 1, adjusted for age, sex, race, education attainment; Model 2, further adjusted for marital status, poverty-income ratio, total energy intake, smoking, drinking; Model 3, further adjusted for arthritis, thyroid problems, cancer, diabetes, depression, CVD, liver diseases.

CI = confidence interval, CMI = cardiometabolic index, CVD = cardiovascular disease, HR = hazard ratio.

**Figure 3.**
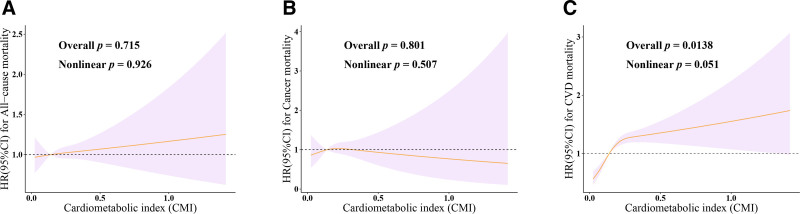
Dose–response relationship of continuous CMI with all-cause mortality and cause-specific mortality. Three knots (10th, 50th, and 90th percentile) were selected for fitting the restricted cubic spline model, and the median value of CMI was used as the reference point. Models were adjusted for age, sex, ethnicity, marital status, education, poverty–income ratio, total energy intake, smoking, drinking, arthritis, thyroid problems, cancer, diabetes, depression, CVD, and liver diseases. (A) All-cause mortality; (B) Cancer mortality; (C) CVD mortality. CI = confidence interval, CMI = cardiometabolic index, CVD = cardiovascular disease, HR = hazard ratio.

We further used a 3-knot restricted cubic spline to estimate the dose–response relationship between CMI and the risk of mortality (Fig. 3). The results further demonstrate a significant linear association between continuous CMI and the risk of CVD mortality (Overall *P* = .0138, Nonlinear *P* = .051).

### 3.3. Subgroup analysis and sensitivity analysis

**Figure 4.**
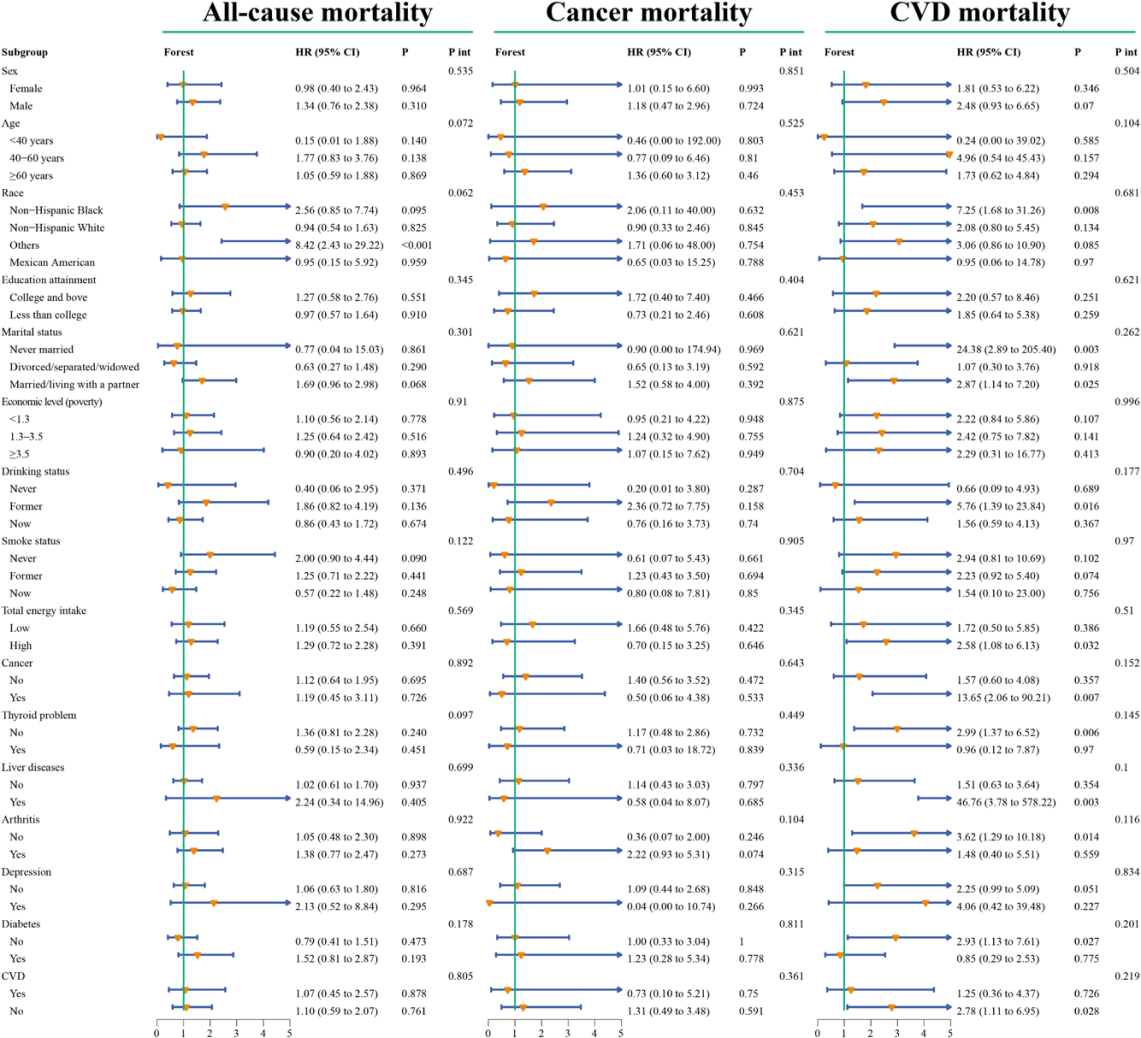
Forest plot of subgroup analysis and interaction tests for the association between CMI and all-cause mortality and cause-specific mortality. CI = confidence interval, CMI = cardiometabolic index, CVD = cardiovascular disease, HR = hazard ratio, *P* int = *P* for interaction.

Subgroup analysis and interaction tests indicated consistent associations between CMI and the 3 types of mortality across all strata (all *P*-values > .05), with no identified specific subgroups (Fig. 4). Sensitivity analysis further strengthened the evidence of our results, as excluding participants who died within 2 years of follow-up, without considering weighting or multiple imputations for missing covariates, showed associations between CMI and the risk of the 3 types of mortality consistent with the main findings (Table S1, Supplemental Digital Content, https://links.lww.com/MD/P552).

## 4. Discussion

To our knowledge, this is the first study to explore the association between CMI and the risk of mortality in a U.S. adult population. In this longitudinal cohort study, the weighted sample represented approximately 80 million U.S. adults aged 20 and above. Survival analysis and Cox regression consistently showed that CMI increased the risk of CVD mortality, while it was not significantly associated with the risk of all-cause mortality and cancer mortality. Trend tests and restricted cubic spline both indicated a linear dose–response relationship between CMI and the risk of CVD mortality. In subgroup analysis, our results suggested a consistent relationship between CMI and the risk of CVD mortality across all strata, with no identified modifying relationships. Finally, several sensitivity analysis, including the exclusion of participants who died within 2 years of follow-up, without considering weighting or multiple imputations for missing covariates, all showed associations between CMI and the risk of the 3 types of mortality consistent with the main findings, thus, further enhancing the credibility of this study’s results.

Although research on the relationship between CMI and the risk of mortality is still lacking, multiple studies have confirmed the association of CMI with metabolic and CVDs. Analyzing 4445 ordinary residents aged 40 and above in northern China, Li et al^[23]^ found a strong and independent association between CMI and stroke. In a study by Wang et al,^[34]^ it was further revealed that CMI had a higher independent association with ischemic stroke in women compared to men. Additionally, a prospective cohort study from Japan, spanning 15.1 years and including 6684 individuals aged 30–79 without a history of CVD, showed that CMI was the strongest predictor of ischemic CVD among TG, TG/HDL-C, and CMI, as reported by Higashiyama et al.^[35]^ Furthermore, a cross-sectional study by Wang et al^[21]^ analyzed 11,400 participants in China and found that incremental CMI was associated with a 35.6% increase in the risk of hypertension in women and a 31% increase in the risk of hypertension in men. Importantly, a cross-sectional study from Japan indicated a strong correlation between CMI and markers of atherosclerosis progression in peripheral arterial disease patients, including intima-media thickness of the common carotid artery and ankle-brachial systolic pressure index, supporting CMI as a reliable indicator for predicting the severity of CVD and CVD mortality.^[25]^

In fact, similar to CMI, indices reflecting lipid metabolism or obesity by combining anthropometric measurements and biochemical parameters, such as the VAI, have revealed associations between metabolic factors and mortality.^[26,36]^ A prospective cohort study from the United States suggested a J-shaped nonlinear relationship between VAI levels in elderly individuals and all-cause mortality.^[12]^ In our study, we found that the association between CMI and all-cause mortality was not significant (*P* = .869) (Fig. 4), and no significant nonlinear association was observed (Figure S1, Supplemental Digital Content, https://links.lww.com/MD/P552). However, the study by Vogel et al^[37]^ indicated that higher VAI in surviving patients with ischemic heart failure was associated with better prognosis and lower risk of death, presenting a seemingly contradictory conclusion that necessitates further detailed subgroup studies on VAI and CVD mortality. Considering the “obesity paradox” and the strong obesity discrimination ability of CMI,^[6,16,17,38]^ further exploration of the risk of death associated with CMI in different CVD populations is also needed in the future. In a study from the UK Biobank, VAI score showed significant linear positive correlations with all-cause mortality risk, cancer mortality rate, and CVD mortality rate, suggesting that the predicted mortality risk by obesity indicators may not be consistent across different populations.^[39]^ In a large prospective cohort study involving 4 million participants in China, the association between VAI and CVD mortality showed a positive linear relationship, an inverse dose–response relationship with cancer mortality, and a U-shaped nonlinear association with all-cause mortality.^[40]^ Based on this extensive prospective evidence, the association between obesity and lipid metabolism indicators and the risk of mortality exhibits notable regional, ethnic, age, and gender differences, with inconsistent or even contradictory results in characterizing CVD mortality and cancer mortality. Therefore, future exploration of these indicators’ relationships with mortality at a larger, international, multicenter level is still needed.

## 5. Advantages and limitations

Firstly, this study fills in some of the gaps in current research. Secondly, all analyses in this study were weighted, representing approximately 80 million American adults. Through weighted Cox regression and Kaplan–Meier survival analysis, we found that CMI significantly predicts the risk of CVD mortality, while it is unrelated to all-cause mortality and cancer mortality. Furthermore, this association feature remains stable across different populations. Finally, combining 3 sensitivity analyses further enhances the credibility of the conclusions. However, some limitations need to be pointed out. Firstly, due to significant regional and ethnic differences in the association between obesity and lipid metabolism indicators and the risk of death, the study sample is limited to the adult population in the United States, which may restrict the generalizability of the results to other populations. Future research in other countries is still needed. Secondly, although multiple potential confounders were adjusted for in the multivariable model, inevitable residual confounding factors may not have been considered. Thirdly, limited by the research conditions, a large amount of declarative data has been adopted in this study. Although these data intuitively describe the current situation, they are deficient in explaining the dynamic relationships among variables. In the future, experimental data will be introduced to enhance the causal inference ability of this study. Lastly, despite the high accuracy of national death registration on which death diagnoses rely, there is still potential for misclassification.^[41]^

## 6. Conclusion

In conclusion, our study establishes for the first time a positive and linear association between CMI and CVD mortality in the general adult population in the United States. This provides some statistical evidence for CMI as a potential indicator for predicting CVD progression and mortality.

## Acknowledgments

The authors thank the NCHS for their efforts in creating the data for the NHANES.

## Author contributions

**Conceptualization:** Caijuan Huang.

**Data curation:** Caijuan Huang.

**Formal analysis:** Lele Chen, Caijuan Huang.

**Funding acquisition:** Caijuan Huang.

**Investigation:** Caijuan Huang, Lele Chen.

**Methodology:** Caijuan Huang.

**Project administration:** Caijuan Huang.

**Resources:** Caijuan Huang.

**Software:** Lele Chen.

**Supervision:** Caijuan Huang.

**Validation:** Lele Chen.

**Visualization:** Lele Chen.

**Writing – original draft:** Caijuan Huang.

**Writing – review & editing:** Caijuan Huang, Lele Chen.

## Supplementary Material
