# Supplementary material for: Association of cardiometabolic index with all-cause and cause-specific mortality among U.S. adult population: A longitudinal cohort study
Source: Medicine (Baltimore). 2025 Jul 25;104(30):e43532. doi: 10.1097/MD.0000000000043532 (PMC12303531; doi:10.1097/MD.0000000000043532)
Supplement: Supplementary file 1 [file medi-104-e43532-s001.docx]

Association of cardiometabolic index with all-cause and cause-specific mortality among U.S. adult population: a longitudinal cohort study

Caijuan Huang^1#*^, Lele Chen^2#^

^#^: Equal contribution

^1^ Department of Hematology, The First Affiliated Hospital, and College of Clinical Medicine of Henan University of Science and Technology, Luoyang, China.

^2^ General Surgery Department of the Southeast Yu Branch of Henan Provincial People's Hospital, Zhumadian, China.

*** Correspondence:** Caijuan Huang

Email: [18567635682@163.com](mailto:18567635682@163.com)

# Supplementary Figure

## Fig. S1: Dose-response relationship of continuous CMI with all-cause mortality among U.S. population with the age above 60 years

Figure legend: Three knots (10th, 50th, 90th percentile) were selected for fitting the restricted cubic spline model, and the median value of CMI was used as the reference point. Models were adjusted for sex, ethnicity, marital status, education, poverty-income ratio, total energy intake, smoking, drinking, arthritis, thyroid problems, cancer, diabetes, depression, CVD, liver diseases.


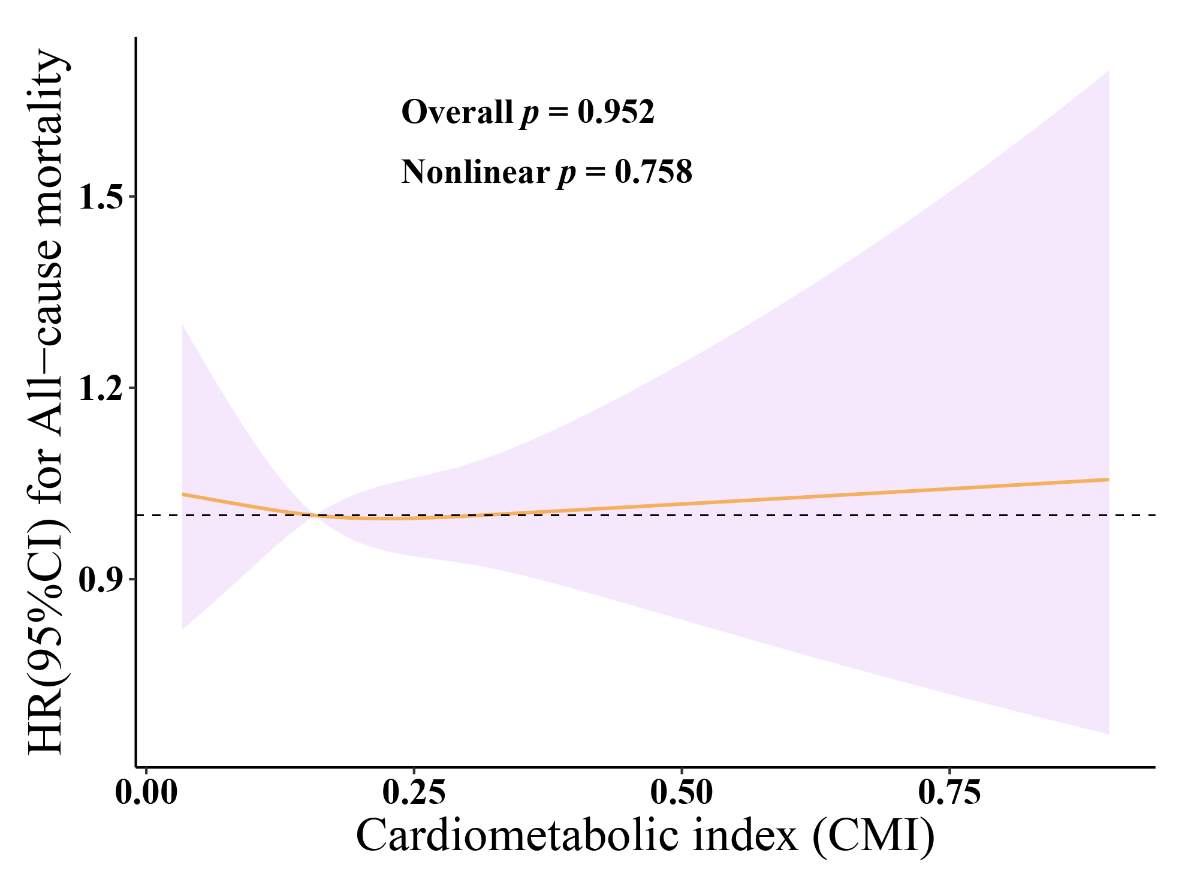


# Supplementary Table

## Table S1: Sensitivity analysis

Notes: Model 0, no confounder were adjusted; Model 1, adjusted for age, sex, race, education attainment; Model 2, further adjusted for marital status, poverty-income ratio, total energy intake, smoking, drinking; Model 3, further adjusted for arthritis, thyroid problems, cancer, diabetes, depression, CVD, liver diseases.

Abbreviation: CVD, cardiovascular disease; HR, hazard ratio; CI, confidence interval.

| **Sensitivity analysis 1: Exclusion of participants who died within two years of follow-up** | | | | | | | | |
| --- | --- | --- | --- | --- | --- | --- | --- | --- |
| **All-Cause mortality** | |  |  |  |  |  |  |  |
|  | Model 0 | | Model 1 | | Model 2 | | Model 3 | |
|  | HR (95%CI) | P | HR (95%CI) | P | HR (95%CI) | P | HR (95%CI) | P |
| Quartile 1 | ref |  | ref |  | ref |  | ref |  |
| Quartile 2 | 1.52(1.19,1.94) | <0.001 | 1.06(0.82,1.37) | 0.65 | 1.04(0.81,1.34) | 0.75 | 0.99(0.77,1.28) | 0.95 |
| Quartile 3 | 1.89(1.48,2.42) | <0.0001 | 1.16(0.88,1.52) | 0.30 | 1.05(0.80,1.38) | 0.72 | 0.95(0.72,1.25) | 0.71 |
| Quartile 4 | 2.39(1.89,3.03) | <0.0001 | 1.32(1.03,1.69) | 0.03 | 1.18(0.93,1.50) | 0.18 | 0.97(0.75,1.24) | 0.78 |
| p for trend |  | <0.0001 |  | 0.01 |  | 0.14 |  | 0.72 |
| Continuous | 3.51(2.43,5.07) | <0.0001 | 2.26(1.47,3.45) | <0.001 | 1.77(1.15,2.74) | 0.01 | 1.14(0.69,1.87) | 0.61 |
| **Cancer mortality** | |  |  |  |  |  |  |  |
| Quartile 1 | ref |  | ref |  | ref |  | ref |  |
| Quartile 2 | 1.29(0.78,2.15) | 0.32 | 0.85(0.52,1.41) | 0.54 | 0.85(0.52,1.39) | 0.52 | 0.89(0.54,1.46) | 0.64 |
| Quartile 3 | 1.50(0.93,2.41) | 0.10 | 0.87(0.52,1.47) | 0.61 | 0.76(0.46,1.26) | 0.29 | 0.77(0.46,1.30) | 0.33 |
| Quartile 4 | 1.89(1.08,3.30) | 0.03 | 1.03(0.59,1.80) | 0.93 | 0.92(0.54,1.58) | 0.77 | 0.93(0.51,1.67) | 0.80 |
| p for trend |  | 0.02 |  | 0.76 |  | 0.85 |  | 0.82 |
| Continuous | 2.00(1.01,3.96) | 0.05 | 1.03(0.38,2.80) | 0.95 | 0.75(0.26,2.11) | 0.58 | 0.73(0.25,2.14) | 0.56 |
| **CVD mortality** | |  |  |  |  |  |  |  |
| Quartile 1 | ref |  | ref |  | ref |  | ref |  |
| Quartile 2 | 3.18(2.21,4.58) | <0.0001 | 1.88(1.28,2.75) | 0.001 | 1.87(1.27,2.75) | 0.002 | 1.69(1.16,2.46) | 0.01 |
| Quartile 3 | 4.06(2.77,5.94) | <0.0001 | 2.18(1.45,3.26) | <0.001 | 1.98(1.31,2.98) | 0.001 | 1.69(1.12,2.55) | 0.01 |
| Quartile 4 | 5.13(3.47,7.58) | <0.0001 | 2.63(1.74,3.97) | <0.0001 | 2.39(1.59,3.59) | <0.0001 | 1.77(1.16,2.70) | 0.01 |
| p for trend |  | <0.0001 |  | <0.0001 |  | <0.001 |  | 0.04 |
| Continuous | 4.54(2.55,8.07) | <0.0001 | 4.13(2.08,8.20) | <0.0001 | 3.74(1.85,7.54) | <0.001 | 2.13(1.03,4.40) | 0.02 |
|  |  |  |  |  |  |  |  |  |
| **Sensitivity analysis 2: Disregarding weights** | | | | | | | | |
| **All-Cause mortality** | |  |  |  |  |  |  |  |
|  | Model 0 | | Model 1 | | Model 2 | | Model 3 | |
|  | HR (95%CI) | P | HR (95%CI) | P | HR (95%CI) | P | HR (95%CI) | P |
| Quartile 1 | ref |  | ref |  | ref |  | ref |  |
| Quartile 2 | 1.58(1.32,1.89) | <0.0001 | 1.12(0.93,1.34) | 0.23 | 1.12(0.93,1.34) | 0.23 | 1.08(0.90,1.29) | 0.43 |
| Quartile 3 | 1.90(1.59,2.26) | <0.0001 | 1.22(1.02,1.46) | 0.03 | 1.18(0.99,1.40) | 0.07 | 1.08(0.91,1.29) | 0.38 |
| Quartile 4 | 2.15(1.81,2.55) | <0.0001 | 1.26(1.06,1.50) | 0.01 | 1.16(0.98,1.39) | 0.09 | 1.01(0.85,1.21) | 0.87 |
| p for trend |  | <0.0001 |  | 0.005 |  | 0.1 |  | 0.91 |
| Continuous | 2.83(2.10,3.79) | <0.0001 | 2.06(1.43,2.96) | <0.001 | 1.64(1.12,2.40) | 0.01 | 1.15(0.76,1.74) | 0.49 |
| **Cancer mortality** | |  |  |  |  |  |  |  |
| Quartile 1 | ref |  | ref |  | ref |  | ref |  |
| Quartile 2 | 1.41(0.99,2.01) | 0.05 | 0.96(0.67,1.37) | 0.83 | 0.98(0.69,1.40) | 0.91 | 1.00(0.70,1.43) | 1.00 |
| Quartile 3 | 1.61(1.14,2.27) | 0.01 | 0.98(0.69,1.40) | 0.93 | 0.92(0.65,1.31) | 0.65 | 0.91(0.64,1.30) | 0.62 |
| Quartile 4 | 1.88(1.35,2.63) | <0.001 | 1.09(0.78,1.54) | 0.60 | 1.01(0.72,1.43) | 0.95 | 0.99(0.69,1.41) | 0.95 |
| p for trend |  | <0.001 |  | 0.49 |  | 0.96 |  | 0.87 |
| Continuous | 2.28(1.19,4.38) | 0.01 | 1.65(0.74,3.69) | 0.22 | 1.28(0.55,2.95) | 0.57 | 1.18(0.49,2.83) | 0.72 |
| **CVD mortality** | |  |  |  |  |  |  |  |
| Quartile 1 | ref |  | ref |  | ref |  | ref |  |
| Quartile 2 | 2.06(1.43,2.97) | <0.001 | 1.27(0.88,1.83) | 0.20 | 1.29(0.89,1.86) | 0.17 | 1.24(0.86,1.79) | 0.25 |
| Quartile 3 | 2.88(2.04,4.08) | <0.0001 | 1.66(1.17,2.36) | 0.004 | 1.57(1.11,2.23) | 0.01 | 1.44(1.01,2.06) | 0.04 |
| Quartile 4 | 3.14(2.23,4.43) | <0.0001 | 1.75(1.23,2.48) | 0.002 | 1.61(1.13,2.29) | 0.01 | 1.44(1.00,2.06) | 0.05 |
| p for trend |  | <0.0001 |  | <0.001 |  | 0.005 |  | 0.04 |
| Continuous | 3.24(1.96,5.36) | <0.0001 | 3.13(1.69,5.78) | <0.001 | 2.81(1.46,5.42) | 0.002 | 2.16(1.06,4.38) | 0.03 |
|  |  |  |  |  |  |  |  |  |
| **Sensitivity analysis 3: Multiple imputation for missing covariates** | | | | | | | | |
| **All-Cause mortality** | |  |  |  |  |  |  |  |
|  | Model 0 | | Model 1 | | Model 2 | | Model 3 | |
|  | HR (95%CI) | P | HR (95%CI) | P | HR (95%CI) | P | HR (95%CI) | P |
| Quartile 1 | ref |  | ref |  | ref |  | ref |  |
| Quartile 2 | 1.61(1.42,1.81) | <0.0001 | 1.06(0.94,1.20) | 0.35 | 1.05(0.93,1.20) | 0.43 | 1.02(0.89,1.16) | 0.80 |
| Quartile 3 | 2.03(1.81,2.28) | <0.0001 | 1.15(1.02,1.29) | 0.02 | 1.17(1.03,1.32) | 0.01 | 1.09(0.96,1.23) | 0.19 |
| Quartile 4 | 2.35(2.09,2.63) | <0.0001 | 1.26(1.12,1.42) | <0.0001 | 1.22(1.08,1.38) | 0.001 | 1.08(0.95,1.22) | 0.22 |
| p for trend |  | <0.0001 |  | <0.0001 |  | <0.001 |  | 0.13 |
| Continuous | 2.93(2.45,3.49) | <0.0001 | 1.91(1.51,2.41) | <0.0001 | 1.69(1.33,2.16) | <0.0001 | 1.29(0.99,1.68) | 0.06 |
| **Cancer mortality** | |  |  |  |  |  |  |  |
| Quartile 1 | ref |  | ref |  | ref |  | ref |  |
| Quartile 2 | 1.63(1.27,2.11) | <0.001 | 1.11(0.86,1.43) | 0.44 | 1.06(0.82,1.39) | 0.64 | 1.05(0.81,1.37) | 0.72 |
| Quartile 3 | 2.11(1.66,2.69) | <0.0001 | 1.24(0.97,1.59) | 0.09 | 1.18(0.91,1.52) | 0.21 | 1.15(0.89,1.48) | 0.29 |
| Quartile 4 | 2.47(1.95,3.13) | <0.0001 | 1.33(1.04,1.69) | 0.02 | 1.25(0.98,1.61) | 0.08 | 1.22(0.95,1.58) | 0.12 |
| p for trend |  | <0.0001 |  | 0.01 |  | 0.04 |  | 0.07 |
| Continuous | 2.80(1.93,4.06) | <0.0001 | 1.65(1.01,2.69) | 0.04 | 1.49(0.89,2.51) | 0.13 | 1.38(0.80,2.37) | 0.25 |
| **CVD mortality** | |  |  |  |  |  |  |  |
| Quartile 1 | ref |  | ref |  | ref |  | ref |  |
| Quartile 2 | 1.84(1.45,2.34) | <0.0001 | 1.15(0.91,1.46) | 0.24 | 1.15(0.90,1.47) | 0.26 | 1.10(0.86,1.40) | 0.46 |
| Quartile 3 | 2.76(2.21,3.46) | <0.0001 | 1.47(1.17,1.85) | <0.001 | 1.46(1.16,1.85) | 0.001 | 1.33(1.05,1.68) | 0.02 |
| Quartile 4 | 3.15(2.53,3.93) | <0.0001 | 1.60(1.28,2.01) | <0.0001 | 1.53(1.21,1.93) | <0.001 | 1.34(1.06,1.70) | 0.01 |
| p for trend |  | <0.0001 |  | <0.0001 |  | <0.0001 |  | 0.004 |
| Continuous | 3.53(2.66,4.69) | <0.0001 | 2.63(1.80,3.84) | <0.0001 | 2.23(1.49,3.33) | <0.0001 | 1.78(1.15,2.75) | 0.01 |
